# Experiences of healing therapy in patients with irritable bowel syndrome and inflammatory bowel disease

**DOI:** 10.1186/s12906-015-0611-x

**Published:** 2015-04-03

**Authors:** Andrew Soundy, Rhonda T Lee, Tom Kingstone, Sukhdev Singh, Pankaj R Shah, Lesley Roberts

**Affiliations:** School of Sport, Exercise and Rehabilitation Sciences, University of Birmingham, Birmingham, B15 2TT UK; Integrated Medicine Department, Freshwinds Charity, Prospect Hall, 12 College Walk, Selly Oak, Birmingham, B29 6LE UK; Department of Gastroenterology, Consultant in Gastroenterology, Good Hope Hospital, Sutton Coldfield, UK; Primary Care Clinical Sciences, School of Health and Population Sciences, University of Birmingham, Edgbaston, Birmingham, B15 2TT UK

**Keywords:** Healing, Thematic analysis, Therapeutic touch, Irritable bowel syndrome, Inflammatory bowel disease, Patient experiences, Complementary therapy, Healing, Irritable bowel syndrome, Ulcerative colitis, Crohn’s disease, Qualitative

## Abstract

**Background:**

The use and value of different complementary therapies requires investigation. In particular, qualitative research is required to understand the perceptions and experiences of patients who undergo healing therapy as one type of complementary therapy. The aim of this research is to consider patients perceptions and experiences following a course of healing therapy.

**Methods:**

Twenty two patients took part in this study. This included 13 patients with irritable bowel disease (3 male, 10 female, 47.6 ± 15.0 years), 6 patients with ulcerative colitis (3 male, 3 female, 48.5 ± 25.6 years) and 3 female patients with Crohn’s Disease (45.0 ± 5.2 years). Each patient undertook a single semi-structured interview following a course of healing therapy. The data was analysed using a thematic analysis.

**Results:**

Three broad themes were identified from patient interviews (1) The understanding and expectation of healing (2) Experiences and reflection on healing (3) Impact and outcome of healing. The details of each theme are explored within the text, often revealing a unique experience of healing therapy.

**Conclusion:**

Patients were open towards the benefits that could be attained by healing, although most patients were not sure what healing would entail. Some patients expected to be relaxed by the sessions. However, the most consistent reports were that patients experienced a relaxing sensation that was generated within the session and lasted for a time period after the sessions. In addition to this the healing appeared to be associated with patients feeling more tolerant of their symptoms. Patients valued the therapist and their input into the healing process. It should be noted however, that this report cannot consider the efficacy of the treatment. Further details and experiences are considered within the article, including one negative experience.

**Electronic supplementary material:**

The online version of this article (doi:10.1186/s12906-015-0611-x) contains supplementary material, which is available to authorized users.

## Background

Irritable bowel syndrome (IBS) is a common functional condition characterised by abdominal pain, bloating and disruption to bowel habit; constipation, diarrhoea and a combination thereof, all being recognised symptoms. These symptoms are often combined with coexisting psychological morbidity including symptom-related fears, anxiety and somatisation [1]. The global prevalence of IBS has been reported as 11.2% [2], although considerable variation is apparent across countries. Within the UK the prevalence has been reported between 6.5% [3] and 10% [4], although not all patients seek medical attention and around 60% of individuals with IBS in the UK may not have been formally diagnosed [3,5]. The diagnosis is one of exclusion and is given when symptoms are present in the absence of abnormal pathophysiology. ROME II and III criteria are typically applied in a research context to enable more accurate categorisation of patients with this disorder. IBS results in reduced quality of life, adversely affecting patients’ general wellbeing, as well as their social, vocational, and sexual functioning [3,6-8]. The treatment options for IBS are limited and for a group of patients (typically those seen in secondary care settings [9]) available treatment may be poorly efficacious.

In contrast Inflammatory Bowel Disease (IBD), which includes Crohn’s Disease (CD) and Ulcerative Colitis (UC) has a recognised organic pathophysiology. It is characterised by similar symptom presentation as IBS but also typically presents with blood stained diarrhoea and weight loss. IBD is less prevalent than IBS and affects around 1 in 1000 (0.1%) of the population [10,11], although higher prevalence in more westernised societies [12]. In the UK around 50,000 individuals has IBD [13]. Despite a much wider range of medical and surgical treatment options, many young adults remain symptomatic and experience poor quality of life [11].

### Healing and complementary and alternative medicine

The use of complementary therapy in the UK is widespread with estimates of up to 20% using such a therapy in the previous year [14]. In England, approximately 90% of complementary or alternative medicine (CAM) is purchased privately by individuals, at a cost of around £450 million [15]. Despite the widespread use of CAM the medical community highlight the dearth of evidence which limits adoption into mainstream medicine [16], and evidence currently available is highlighted as being of poor quality [17], meaning the evidence has been described as inconclusive [18]. The public demand combined with lack of evidence often leads to adoption of untested therapies by National Health Service (NHS) trusts or significant personal investment of patients trying to seek benefit outside of the NHS.

Healing therapy, also known as ‘Therapeutic touch’, is one such therapy with increasing use, but has a very restricted evidence base. Therapeutic touch is based on eastern philosophy and is based on the premise “*that spiritual aspects of oneself (the inner self) inform and guide the physical self, and may direct the conditions of one’s life*” [19] (p. 78). The idea of therapeutic touch is to influence an individual’s vital energy field. The concept is based on the premise that humans are infused with subtle forms of energy or biofields [20,21]; a concept which is putative as such energy fields have yet to be quantified [22]. Therapeutic touch healers are trained to identify problem areas, like fatigue and pain, and identify other signs of imbalance [23]. The impact of therapeutic touch is based on “*a cause-and-effect relationship between a healer’s conscious intension to heal and subsequent improvement in client symptoms*” [4] (p. 174). Therapeutic touch takes place in a calm and peaceful area by a trained therapist.

### Qualitative research and healing

Previous research and reviews of research have provided rich accounts of the experiences of patients with IBS and IBD [24-30]. These studies have identified the value of qualitative research in describing the personal expressions of patients with IBS and IBD and the significant impact physically and psychosocially these illnesses have. In contrast to this, research considering the use of therapeutic touch or healing therapy in this patient group is limited [18,20] and further research examining the personal experiences of patients undergoing such treatments would be beneficial. Indeed, whilst there is a need for good quality quantitative research documenting any effect of healing on outcomes such as symptoms, quality of life and healthcare utilisation [18], the use of outcome measures can vary limiting comparison between studies and the sensitivity of instruments used may mean that some outcomes are not captured [31]. In essence, the current use of quantitative outcome measures would benefit from data derived from qualitative methods in supporting the evidence for efficacy or introduction of a treatment. The same need for qualitative research has been identified in other chronic illnesses that have considered healing as a therapy [32].

The aim of this study was therefore to explore the experiences of healing therapy in patients with IBS and IBD. The objectives were threefold; (a) to consider patients perception of healing, (b) to consider the experiences during the session, and (c) to identify the patients perception of the impact of the healing sessions on symptoms.

## Methods

### Participants

Twenty two patients, 13 patients with IBS (3 male, 10 female, 47.6 ± 15.0 years), 6 patients with UC (3 male, 3 female, 48.5 ± 25.6 years) and 3 female patients with CD (45.0 ± 5.2 years) who were engaged in a larger randomised controlled trial of healing therapy took part in this study. Table 1 presents demographic details of patients.

**Table 1:** **Providing demographic details of participants**

**Participant**	**Age**	**Time with condition**		Employment status*
**Participant/gender/condition**	**Age (mean)**	**Length of time with condition (yrs)**	**Length of time with diagnosis (yrs)**	
**P1 M IBS**	39	10	1.5	NW
**P2 F IBS**	18	1.1	0.5	PT
**P3 F IBS**	45	2.5	0	NW
**P4 M UC**	77	30	30	R
**P5 F UC**	71	13	13	R
**P6 F IBS**	43	15	15	NW
**P7 F IBS**	57	5	5	NW
**P8 F IBS**	45	20	12	NW
**P9 F UC**	22	1	0.3	NW
**P10 M UC**	28	13	13	PT
**P11 F IBS**	54	5	5	NW
**P12 F UC**	26	4	4	NW
**P13 F IBS**	67	2	0.25	R
**P14 F CD**	39	14	4	NW
**P15 F IBS**	51	1.5	1.5	FT
**P16 M UC**	67	30	30	R
**P17 F CD**	48	7	7	PT
**P18 M IBS**	53	0.5	0.1	FT
**P19 F CD**	48	25	25	PT
**P20 F IBS**	56	10	10	PR
**P21 F IBS**	69	15	2	R
**P22 M IBS**	22	5	5	PT
**Mean**	**47.5**	**10.4**	**8.4**	
**(SD)**	**(17.0)**	**(9.1)**	**(9.4)**	

Note: *FT = full time, PT = part time, R = retired, NW = not working.

### Eligibility criteria

Within the feeder trial (ISRCTN: ISRCTN13039379, DOI: 10.1186/ISRCTN13039379) all patients were aged 18 and above, receiving outpatient care at a single NHS trust in the West Midlands region of the UK with a clinician diagnosis of IBS (confirmed by ROME II criteria) or UC or CD. Patients already receiving healing therapies (or having done so in the previous 6 months) were excluded. Patients unable to give fully informed consent were excluded. In line with clinical trial guidelines pregnant women were not included and individuals currently engaged in any other clinical trial or having completed such a trial in the previous 8 weeks were excluded.

For the purposes of this study any individual who participated in the larger trial and had received healing therapy as a trial intervention was eligible for selection. Selection of patients for the qualitative element of this work was undertaken purposively [33] to capture a range of diagnostic and demographic characteristics.

### Design and intervention

In the feeder trial, all patients undertook five weekly sessions of 30 minutes (as suggested by a pilot study of 183 patients with IBS/IBD focusing on the feelings and experiences following a single 20 minute healing session, this was combined with the opinion of the current researchers and healing therapists used in this study) of healing therapy (an energy therapy) [16] either immediately after randomisation or after a waiting list control period (this did not impact the study findings as both groups received healing). Therapy was delivered by trained therapists at two hospitals in the midlands of England and was in addition to usual clinical management. Therapy was individually focussed (as in usual practice) and normally involved the patient being in a lying down position. The environment was a single quite room within the Gastroenterology clinic where the healing took place, the room had a plinth and depending on the therapist, music may have been played to promote a relaxed atmosphere, this was to ensure the ecological validity. The therapist commenced the treatment usually starting from the head placing their hands at a distance of about 10–12 inches above the body. Light touch was used (with participant permission) on occasion based on the therapists judgement and included gentle holding of the sides of the head, shoulders, hands or feet for short periods of about 2–3 minutes to enhance the healing treatment. For further details please refer to the healing trust website (http://www.thehealingtrust.org.uk/about/healing, retrieved 30/03/2012).

### Healing and therapists

Healing therapy was delivered by practicing members of the Healing Trust (HT), which identifies and defines spiritual healing as a ‘natural energy therapy’ that their healers use, acting as a ‘conduit for healing energy’ (www.thehealingtrust.org.uk, retrieved 30/03/2012). The healing therapy provided for this study was only that defined by the trust, rather than broader or other definitions healing e.g., Reiki. Within the field of CAM, there are a number of approaches to Healing. By restricting our choice of Healing therapists to members of the Healing Trust, we ensured consistency of the Healing approach within the trial. We also could be assured that the healer would have a specific standard of training and would adhere to a professional code of conduct. The HT was established in 1954 and is the largest healer membership organisation in the UK with over 4,500 members. The HT is a pan-denominational organisation that promotes healing in over 50 voluntary healing centres throughout the UK.

### Interview

A variation of patients was selected to include a range of ages, gender and employment together with similar numbers from the intervention or waiting list control group. Patients were interviewed immediately following their fifth and final healing session. The interviews were arranged via a face to face meeting with the research assistant author (TK), at which point an information sheet was provided. No prior relationship was established with patients by author AS, nor was any personal biases identified to patients before the interview. The digitally recorded interviews (lasting between 23–67 minutes, mean 36 min) took place in a quiet room within Gastroenterology clinic, next to/near by the rooms allocated for healing. The primary author (AS) has previous experience of interviewing patients with chronic illness [34,35] and undertook all interviews. Before the interview began the reason for undertaking the interview was explained to patients, and, for the purpose of ethics, patients were told that at any time the interview could be stopped and their data removed with no subsequent influence on or to their care. No adverse effects were reported and no patient asked for their interview to be stopped or data removed from the study. The interview schedule was based on our pilot research, previously published research [20] and the opinions of a multi-disciplinary and experienced research group which included a specialist in healing, a consultant for a charity specialising in CAM, a consultant in gastroenterology, academic researchers and a CAM practitioner. Interviews included open ended questions relating to perceived needs of individuals, the experience of therapy and perceived effects of intervention. The interview schedule was adapted following the development of the third thematic framework (see below) and the first ten interviews based on initial analysis. The final interview schedule is provided in Additional file 1.

### Analysis

For the purpose of the study and analysis the primarily author was positioned as a ‘subtle realist’ [36], as such an approach to analysis was selected which reflected this position. Thus, whilst the importance of unique experiences from patients was recognised, the analysis was designed to identify common or shared experiences and perceptions across the whole group of patients. For this purpose a thematic analysis [37] was conducted on the data generated. This type of analysis is good at identifying the salient issues of a particular group of respondents [38]. All interviews were transcribed verbatim and analysed by the primary author, who then transcribed the data and conducted the analysis, providing the initial thematic development (themes 1 and 2; see Table 2). The process of this involved a constant target comparison [39], fragmenting the data [40] and idea webbing [41] for thematic development. This analysis followed the first 10 interviews. After this a copy of the themes (see Table 2) was provided to five of the co-authors. Their role was to consider the analysis in relationship to the transcribed data, acting as ‘critical friends’ [42,43]. Each author reviewed two interview scripts and considered how these related to the thematic development and commented on the appropriateness of the theme, and also considered whether themes had been overlooked [44]. Following feedback a third thematic framework was developed (themes 3; see Table 2) the new interview schedule was developed, the reason for this was to focus on, and saturate the result themes by making the schedule reflect the thematic development in order to explore it in greater depths. The thematic framework was further revised (themes 4; see Table 2) and finally narrowed down to three broad themes (themes 5; see Table 2). Further details can be obtained from the primary author. An example of analytical techniques is provided within a Additional file 2.

**Table 2:** **The thematic development illustrating key stages when themes were changed (details and content from each stage is available from the primary author)**

**Themes 1**	**Themes 2**	**Themes 3**		**Themes 4**	**Themes 5**
Descriptions of acute change after sessions	Understanding of illness and healing	Bio psychosocial impact and outcomes of healing	New Interview Schedule Generated (see Additional file 1)	Perception of Healing	Understanding and expectations of Healing
Descriptions of acute change during sessions	Preparation for sessions	Bio psychosocial impact of illness on patient		Bio-psychosocial impact of illness on the patient	
Symptom changes	Immersing in the Healing environment	Preparation, experiences and reflection on healing		Experiences and reflection on healing	Experiences and reflection on Healing
Factors attributed to the illness	Transference			Impact and outcome of healing	Impact and outcome of Healing
Cognitive changes					
Searching					
Perception of healing					
Getting used to healing					
Understanding the disease					

### Ethics

Ethical approval was obtained from the National Research Ethics Service. The study was reviewed by The Black Country Research Ethics Committee. The research ethics committee approval number was 10/H1202/36. Date of study approval was 17/06/2010.

## Results

Three broad themes (see Table 2, themes 5) were identified from the patient interviews: 1) The understanding and expectation of healing, 2) Experiences and reflection on healing, 3) Impact and outcome of healing.

### The understanding and expectations of healing

Six patients said they didn’t know what was meant by the term ‘healing’ (in relation to, or as a therapy within CAM) before they entered the trial. Five patients specifically stated they had a recommendation from others (such as friends) regarding the trial. For example, one patient stated *“I have known other people have healing…and got good results”* P18 M IBS. Five patients stated they had previous knowledge or experience of healing. The expectations of what patients would get from healing, most frequently, did not involve a cure. Only two patients before the trial specifically said they thought it would benefit their symptoms, although four patients expected the healing sessions to be relaxing. By virtue of their participation in the study patients were willing to experience healing therapy and four patients mentioned this open minded viewpoint, for example, “*I mean I am quite open minded about things really…it may help with my stress situation*” (P16 M UC). Similarly, five patients reported having no prior expectations. Four patients were initially sceptical about it, whilst four stated they felt it may help them be more relaxed. Table 3 provides details and further examples of the subthemes.

**Table 3:** **The understanding and expectations of patients pre-trial**

**Theme**	**Sub-theme**	**Code**	**Examples from patients**
**Understanding of Healing**	Little understanding		“I didn’t really know what I was going into” P1 M IBS
			“I don’t know [anything about healing]” P2 F IBS
			“No, the only thing was what I read in the mail [newspaper]” P16 M UC
	Awareness of healing	Recommendation	“I have worked with a girl that used to be a beautician and she suggested different things…when Dr [name] mentioned it, I thought I would like to try it” P12 F UC
		Previous knowledge or experience	“I am in the industry myself and that helps” P10 M UC
			“I have done Reiki in the past” P18 M IBS
			“I have had Reiki once before” P20 F IBS
**Expectation of Healing**	Scepticism		“I must admit I was pretty much sceptical about it” P1 M IBS
			“I didn’t think it could heal you or anything like that” P19 F UC
			“Being realistic I know that nothing is going to cure it” P17 F UC
	Unsure of what to expect	No idea	“I didn’t know what to expect” P2 F IBS
			“I didn’t really know, know what to expect before” P6 F IBS
			“I didn’t know what to expect” P13 F IBS
			“I wasn’t hoping it would heal me because that would be a miracle” P14 F UC
	Openness	Open to it	“I came in with an open mind” P19 F CD
			“I went into it with an open mind…because I just wanted to see what would happen” P20 F IBS
	Some benefit	Being more calm or relaxed	“It has been [being relaxed has been achieved]” P5 F UC
			“I think my stress may be [helped]” P16 M UC
		Sleep better	“To sleep a bit better than I did” P13 F IBS
		Hope that it could help	“If there was a benefit then it would help to relieve the symptoms” P18 M IBS
			“What I really wanted was to come off the tablets” P21 F IBS
		Was told it would be beneficial	“She [a friend who was a therapist] said you need healing a lot” P15 F IBS

### Experiences and reflection of the healing sessions

Patients described a very positive relationship with their healer which was based on trust and feeling relaxed and comfortable with the person. Three patients identified that therapists were able to make them feel special, through valuing them and giving them time, and through what they said and the way they said it during the sessions. For example, one patient stated “*she made it personal to me, I mean she cradled my head at one point and that felt really nurturing…I found that very emotional*” (P3 F IBS). Four patients described a positive experience through being exposed to the empathy of the therapist.

Patients described different experiences during the sessions. Ten patients said the sessions made them feel more relaxed or calm. Seven patients identified feeling a localised warmth or heat generated from around the therapist’s hands, although this could occur with no contact. Other experiences during healing were very individual to the patient. For example, two patients reported being uplifted by the experiences, two patients reported seeing bright colours and light and one patient identified a sense of energy. One patient experienced visions “*a vision of somebody in the past…I have seen me mum [within the session]…you might see things in your eyes… you will probably see little animals, like this jumping out at you*” (P9 F UC). Additional feelings were identified by patients: two patients described experiences of being sucked or pulled; three patients stated they experienced nervous reactions (e.g. a spasm) and one patient identified a feeling that wind was being chased around her body. Two patients experienced pain during or soon after therapy, which were short lived and the patients did not seek medical intervention. Pain was also identified by one patient who received healing previously. Table 4 provides details and further examples of the subthemes included.

**Table 4:** **Experiences and reflection on healing**

**Theme**	**Sub-theme**	**Code**	**Examples from patients**
**Interaction with the therapist**	Connection	Empathy, comfort and trust	“You need to get comfortable with the lady who is doing it” (P7 F IBS)
			“I had to feel comfortable with the person and that can take a couple of sessions” (P14 F UC)
		Special feeling	“The contact [connection] is a big part of it, a big part of the help and you wouldn’t get that with a psychologist” (P3 F IBS)
			“Made me realise that I am not alone in what I am feeling” (P3 F IBS)
	Communication	Transferring feelings	“The whole wording, what she’s [therapists] talking about” (P1 M IBS)
			“As [therapist] is talking I am aware that she is holding her hands over me or touching parts of my body, reinforces what she is saying” (P11 F IBS).
		Procedure for undesired symptoms experiences	“I don’t know where it came from [the pain] but, they [therapist] did explain it was like a release” (P9 F UC).
			“She [therapist] did tell me that I should rest that evening. You know, not try to do too much…” (P20 F IBS)
			“The next week she [therapist] said ok, we won’t be so intense this time, and she still did some of the Reiki with me, but I sat up” (P20 F IBS)
**Experiences during sessions**	Vivid experiences	Relaxed	“You feel all relaxed and calm” (P4 M UC)
			“I can feel myself relaxing into it” (P7 F IBS)
			“It is its wonderfully relaxing” (P21 F UC)
		Calmer	“I am really a much calmer person” (P6 F UC)
			“It makes you really calm” (P8 F IBS)
			“I feel really calm” (P14 F UC)
		Heat/warmth	“Because the heat is strong” (P3 F IBS)
			“Today I could feel the heat” (P5 F UC)
		Uplifted	“Had a real spring in my step…uplifted…I feel happier” (P14 F UC)
			“I am like a spring chicken the next day, I am just running and racing around” (P15 F IBS)
		Light and energy	“In the day time you have these colours going on” (P1 M IBS)
			“Brighter, I know my eyes are closed and it would actually feel brighter” (P12 F UC)
		Visions	“You might have a vision of somebody in the past which I did…I have seen me mum…Odd things might happen like you might see things in your eyes… you will probably see little animals, like this jumping out at you, and around” (P9 F UC)
		Being pulled or sucked	“Strange experience to be held by the feet and have this kind of trip, it feels you are being pulled through a tunnel” (P1 M IBS)
			“You feel like you’re being hurtled” (P1 M IBS)
		Nervous sensations/reactions	“Feel a bit tingly in my hands and elbows” (P14 F UC)
			“When she touches me stomach their I jump…it’s a reflex action…she was worried about it…nowhere else” (P16 M UC)
		Wind	“You can feel the wind going round in your body…it was like she was chasing wind round my body” (P7 F IBS)
	Undesired experiences	Increased pain	“After the first one [session], it sort of brought all my wind, just under my back and it was quite painful” (P7 F IBS)
			“The first session I was out of control, and the pain I felt and it was, I was just crying…” (P 9 F UC)
		Heat pain	“I had this burning pain which was saying to [therapist] about which was right in the back of my head, really really hot…I had to change my day at work the next day because I still felt really hot as well” (P20 F IBS)
		Crying	“Crying all the time [first session]” (P9 F UC) “when I left the [place of work] the first time [after the first session] I went into the loo and I picked up a, a nice text from my daughter and I just burst into floods of tears” (P20 F IBS)
	Engaging with the experience	Difficulty getting into the breathing	“It was quite difficult to get into with my breathing” (P12 F UC)
			“For the initial session I found if really difficult to focus on me breathing” (P14 F UC)
**Experience following session**	Increased bowel related symptoms		“A good three days after I was continuously windy, I didn’t think anyone could burp or trump that much” (P7 F IBS)

### Impact and outcomes of healing

Nine of the twenty two patients commented on a perceived physical benefit and eleven on a benefit which we have classed as psychosocial.

### Physical impact

Nine patients attributed varied positive changes in their symptoms to the therapy, for example, “*I am going to the toilet more regularly without any medication*” (P7 F5 IBS). Indeed, the most commonly indicated improvement was being able to undertake a more ‘normal’ routine for example, the number of visits to the toilet each day. Three patients reported an improvement in bloating and four identified pain reduction e.g. “*the pain is a lot easier, you feel like doing more stuff and you are not in pain all the time…. It [healing] has mainly helped the pain*” (P8 F IBS). One patient reported that whilst she continued to experience episodes of vomiting these were less severe after healing. Four patients specified that better quality sleep occurred after the healing sessions e.g. “*The sleeping is a big benefit you know …. You feel as though you have had a more satisfying sleep*” (P5 F UC). No patient stated that their symptoms had completely gone. There were a number of other factors which patients indicated which may have influenced the outcome of healing e.g. “*I haven’t had the swelling that I have been having …. I don’t know whether it was because of healing or because I have change my diet*” (P13 F IBS) and “*I also have been on an antidepressant medication, so I think those three [CBT, medication and Healing] things at the same time have been perfect*”; a full list of these other influencing factors are reported in Table 5.

**Table 5:** **Impact and outcome of healing**

**Theme**	**Sub-theme**	**Code**	**Examples from patients**
**Physical Impact**	Improvement No or limited change	More “Normal” Routine	“I am going to the toilet more regularly without any medication” (P7 F IBS)
			“I have been sleeping better…Swelling seems to have gone down” (P8 F IBS)
			“Interviewer: in terms of your bloating, had it stopped your bloating? P15 F IBS: I don’t get it as much as I used to” (P15 F IBS)
		Less Pain	“I haven’t had or haven’t been getting the stomach cramps or diarrhoea as much…it’s probably been better than what I expected” (P2 F IBS)
			“Last week [session 4] I felt so much better…I can actually go to the toilet without too much pain now” (P7 F IBS)
			“The pain is a lot easier you feel like doing more stuff and you are not in pain all the time…it [healing] has mainly helped the pain” (P8 F IBS)
			“I would say the pain is reduced, due to the relaxation it has taught me” (P 22 M IBS)
		Better Sleep	“The sleeping is a big benefit you know…you feel as though you have had a more satisfying sleep” (P5 F UC) “I actually sleep very well from it” (P3 F IBS)
			“[After the 4th session] I slept absolutely brilliantly that night” (P5 F UC)
			“I have noticed that since I have been doing the trial have been sleeping better” (P8 F IBS)
			Interviewer: would you tell me the best and worst part of the therapy? P15 F IBS:…going to sleep [at night]” (P15 F IBS)
			“I can’t see that anything has actually changed” (P1 M IBS)
			“I wouldn’t say it has helped the IBS…I was perhaps hoping, perhaps over time it might” (P11 F IBS)
	Unable to determine		“Interviewer: because you were feeling great you can’t say whether it has done anything at all? P12 F UC: No I can’t.” (P12 F UC) “When I started I was feeling great the best I have ever felt since starting the trial” (P12 F UC)
			“If you are going to ask me questions as to whether I am feeling better, the answer is I don’t know.” P16 M UC
	Other factors influencing change	Medication/Medical Reason	“The stomach cramps were stopped anyway, because of the tablets I have been put on” (P2 F IBS)
			“Every time I plan something [going out, undertaking an activity] I have to have tablets as well” (P2 F IBS)
		Diet	“I haven’t had the swelling that I have been having…I don’t know whether it was because of healing of because I have changed my diet” (P13 F IBS)
		Other Therapy/Activity	“Tuesday which is like a therapy group for people who have panic attacks and anxiety” (P14 F UC)
			“Going to [teaching location] I think has been very positive” (P11 F IBS) “I have done CBT, I have been doing for a while which has been going through the same time” (P22 M IBS).
		Change in Lifestyle/Stress	“There is a slight problem in that I stopped work [extremely stressful position] and went through the healing at around about the same time…[also] I missed 1 [session]” (P18 M IBS) “Any kind of noise outside is a distraction” (P1 M IBS)
		Intensity of healing	“The next week she said ok, we won’t be so intense this time…So I hadn’t felt anything really once she turned the heat down” (P20 F IBS)
		Advice/therapy from healer	“She [therapist] is giving me things to think about different ways to think about” (P11 F IBS)
			“At night if I can’t sleep then I put on the tape [given by therapist] and just listen” (P13 F IBS)
			“So I have to say I am not sure about the Reiki but the counselling, that…has been positive” (P20 F IBS).
		Harder to carry the effects over	“Week 2 or 3 [of the sessions] I was kind of like losing the strategy of how to get back to that sort of zone” (P14 F UC) “a lot of my stress has been lifted after a session and I feel more confident as well, but after a few days I would go back to flat” (P14 F UC)
	Undesired symptoms	Adverse experience	“I wasn’t expecting to feel so bad…I haven’t found it a pleasant experience…I just felt so ill after it [the first session]” (P20 F IBS).
			“The first week…I can’t say if it is connected with the healing I went to my brothers house…and I knew I was going to be sick and I was sick.” (P11 F IBS) “After the third one [session] I ended up in hospital [patient suffered a transient ischemic attack]” (P7 F IBS).
		Unusual experiences	“The first time…I was like totally zombified…my husband took me home and I slept all afternoon and half the evening” (P17 F UC)
		Tiredness	“Every other week it has left me tired, [I am] tired anyway” (P11 F IBS)
			“I quite often get tired, but I yawn, constantly the whole of the time [within the session] when I go home, in the car I am yawning, yawn” (P21 F UC)
**Psychosocial Impact**	Approach to symptoms	Control over condition	“When I feel myself going that way I will make myself relax” (P7 F IBS)
			“How you deal with things, your choices and your reactions are far more relaxed laid back than they would have been before” (P22 M IBS)
		Confident in their ability to cope	“I went to sleep with the cramps and thought, oh well, this happens doesn’t it? Whereas before I would literally be sweating bags” (P1 M IBS)
			“I think if you have a negative mind that is detrimental” (P1 M IBS)
		Less anxiety & thinking	“I am just trying to change my think patterns” (P6 F UC) “Try to think about things differently” (P11 F IBS)
			“It’s making me switch my mind off” (P14 F UC)
		Calm	“It’s made me more calmer inside” (P6 F UC)
			“It’s quite calming” (P12 F UC)
		Positive	“My perception towards my elements has shifted…I’m definitely positive about it…that it is all very positive” (P1 M IBS)
			“They [symptoms] don’t seem to get me down as much” (P1 M IBS)
		Rejuvenated and Alive	“Starting to get back on track” (P10 M UC)
			“I feel a bit cleansed” (P14 F UC) “It’s made me more alert” (P20 F IBS)

Although many patients reported positive outcomes from healing there were undesired outcomes for some patients. One patient reported that the therapy made her feel ill “*I wasn’t expecting to feel so bad…I haven’t found it a pleasant experience…I just felt so ill after it*” (P 20 F15IBS). Extreme fatigue was also reported after healing sessions e.g., “*the first time…I was like totally zombified…my husband took me home and I slept all afternoon and half the evening he kept saying to me are you sure you haven’t been drugged? Or anything*” (P17 F UC). And other patients reported feeling more emotional or tearful after therapy “*when I left [hospital] the first time [after the first session] I went to the loo and picked up a nice text from my [close relative] and I just burst into floods of tears*” (P20 F IBS).

Five patients reported that they could not see any benefit of therapy and a further two patients were not able to determine what it had achieved. Four patients suggested more sessions were needed, to allow participants time to get used to what a healing session included, for example, knowing what to expect or how to relax. This was perceived as a way to ensure optimal benefit from the healing was achieved.

### Psychosocial impact

A psychosocial impact of healing was identified by patients as the most consistent outcome from the healing sessions. Eight patients cited greater confidence in their ability to cope with their condition or have a perception of control over the symptoms as they occur, for example, “*I went to sleep with the cramps and thought, oh well, this happens doesn’t it? Whereas before I would literally be sweating bags*” (P1 M IBS) and “*when I feel myself going that way I will make myself relax*” (P7 F IBS). This ability to cope resulted in less anxiety and it meant that a more normal routine could be established. This was associated with other statements for example, six patients stated they were trying to be less anxious or change what they worry about following healing. In a similar way four patients said they felt calmer. Finally three patients stated they felt more positive and four stated they felt rejuvenated, getting back on track or more normal. For example, “*It’s made me more alert and like a spring chicken*” (P15 F IBS). This may well have been associated with a perceived change in autonomy and openness in trying to change, adapt and consider different ways forward. For example, “*I am going to lead a bit more of an independent life, not dependent on toilets*” (P7 F IBS). This is an interesting outcome given that healing therapy unlike other psychotherapeutic therapies does not explicitly facilitate shifts in autonomy or thought patterns.

The most consistent finding reported by patients was feelings of being relaxed following the sessions. These comments were often qualified by identifying the deep state of relaxation that was achieved. For example, “*I am actually almost as if I could go to sleep, it is a nice sort of relaxing feeling*” (P13 F IBS). In a similar vein, patients identified that they felt calmer in themselves. Table 5 provides details and further examples of the subthemes occurring under the psychosocial impact theme.

## Discussion and conclusions

Nested within our clinical trial to determine effectiveness of healing therapy in IBS and IBD we undertook this qualitative study to explore the healing experience. We sought to learn more of patient’s expectations of healing therapy, their experience of the healing session and to give patients an opportunity to express in their own words the effects of therapy, as they had experienced it. We found that a number of patients were unsure of what healing entailed or what benefits were associated with it. Our results suggested that healing therapy was experienced in a highly individual way, however this qualitative report cannot report on efficacy; this will be reported with the full trial. There were no universal experiences reported by patients, although some consistency was identified in the experiences of being relaxed within the session and feeling more confident in the ability to cope with the impact of symptoms. There were undesired experiences reported by patients, including increased pain and tiredness and one patient (P20 F IBS) reported healing to be a very negative and painful experience for her (details are provided in Additional file 3). The discussion will now focus on the three main themes and outcomes generated.

### Understanding and expectation of healing

There were patients that reported no change in their symptoms and others who reported improvement but were cautious in their reporting and noted that this may be attributable to other factors. It is possible that quantitative outcome measures were not able to detect this paradox, certainly the sensitivity and use of tools has been previously questioned by research [31]. Where symptom changes attributed did occur these were primarily but not exclusively positive. However, this study did not aim to consider the efficacy of healing but to document the range of patient experiences, the evidence of which can be used to provide clarity and detail to the quantitative findings generated by the main study.

### Attitudes towards CAM

Two patients were sceptical but still willing to participate in the trial. This illustrated a consistent ‘openness’ towards trying healing, which was also expressed by other patients on the trial. This ‘openness’ appears to be an important characteristic of individuals who have not used CAM before [45]. Previous research has identified that several other characteristics have been associated with trying CAM treatments. For example, dissatisfaction with conventional medicine [45,46], income [45,47] or a lack of scientific evidence [48]. In a similar way, those who use CAM more often have varied reasons for doing so, for example, for a medical need or as a treat/for enjoyment [49]. In summary, the current study must acknowledge that those who agreed to take part may well hold different characteristics and attitudes than non-participants.

### Impact of healing

Being able to challenge how the symptoms in IBS and IBD are perceived is very important. The unpredictability of patient symptoms (e.g. bowel urgency, faecal incontinence, or cramps) experienced in both IBD [27] and IBS [28,29] means that the symptoms impact and influence every aspect of the patient’s life and lifestyle. Following the healing sessions some patients in the current study were able to take more responsibility for their treatment and were more tolerant of, or were more able to cope with their symptoms. This change directly influenced their ability to undertake a lifestyle of choice and re-engage in social situations, aspects that had suffered previously. The feeling patient’s expressed towards responsibility for their own treatment is important because this illustrates a sense of empowerment [50]. In a similar way engaging in a personal battle for control over the symptoms (especially during an increase in symptoms) is a factor that many patients identify with [27,29,30,51].

One of the most consistent findings from the current research is that patients had time to relax and feel calmer, and this may have influenced their ability to cope following the healing sessions. This is important because patients with IBS often consider that their symptoms are related to stressful situations [50] and during stressful situations they report pain more readily and have reduced coping ability [6,30]. It is worth noting that research in different patient groups have identified an improvement in mental health [52] and anxiety [53] and this literature has acted to support the notion that some benefit on a patient’s health-related quality of life is possible [18]. Previous research has suggested that patients with IBS perceive that they could control their symptoms if they could alter their stress levels [50]. The role of psychological stress in IBD is uncertain [54] and the degree to which patients with IBD feel they can control their symptoms is questionable because often the impact of manipulating diet or stress is uncertain [27].

It is important to consider the individual nature of healing when interpreting these findings for several reasons: (1) some patients reported no or limited change (2) other patients could not determine the change. No significant change has been documented in other research using healing touch e.g., [55] (3) Finally a number of other factors may have influenced the results. For example, a number of patients reported changes in diets or medication during or around the healing therapy, and patient’s identified different interaction with therapists. Indeed some research has considered the possible impact of the illness itself, the setting where the therapy takes place, the patient, therapist or their interaction may have an influence on the outcome [56].

### Experiences of healing

Research suggests that cost and worry of the credential of therapists prevent some patients from seeking therapy [28]. In our study, the willingness of individuals may have been enhanced because of the association of the project with the hospital, University and a CAM charity. Patients in the current study identified the importance of the therapeutic alliance with the therapist as something that was essential to the healing experience. Indeed it is regarded as central in facilitating healing, where the relationship requires collaboration and trust [57].

Feelings of heat and warmth, as well as feelings of being relaxed or calm during healing were common in our patients and have been reported in previous research [58-60]. Other experiences reported in this study have not previously been defined in the literature. Sensations such as being pulled or sucked and the experiencing of visual imagery appear to be highly personal experiences of healing.

There is little known about the contraindications of healing [4]. Previously [61] patients with chronic diseases using healing therapy have reported light-headedness, dizziness, or irritability; these reports may be similar to some of the effects reported by P20 F IBS the following day. The current study revealed some undesired symptoms experienced by patients such as extreme fatigue or tearfulness that was reported to be self-resolved within a few days. Such a phenomenon is consistent with previous research describing short term reactions after receiving complementary therapies [62], and being compared to a process of catharsis and detoxification [63]. As part of patient safety protocols these reactions should always be highlighted to patients before engaging in therapy. Further research considering how the therapists interact, use, report and the advice they give to patients who report undesired symptoms would be beneficial. In addition, it may be worth considering if such experiences are given as reasons why patients have dropped out of research studies. Alongside the potential benefits of healing therapy, and like any other therapeutic intervention understanding the safety of this therapy is essential [18], as it allows clinicians to make informed decisions on its use and role as part of patient care.

### Limitations

The main limitations of this study is that it induces a relatively small sample and may be biased by only including those who agreed to participate in a trial of healing therapy. Therefore, the current results may hold less negative views than would be represented in the wider population. Although the authors feel that whilst data saturation was achieved, the position taken for the purpose of the analysis meant that the highly personal experience of healing may have been limited. Despite inherent bias this population did include individuals who considered themselves sceptical prior to treatment. The study aims only to explore patient experiences of a therapy and cannot explore efficacy. The number of healing sessions undertaken may have influenced patients response and interviewing following a variable number of sessions may be useful. Further the location in the hospital and the environment around that location may have influenced the findings. The interview guide may be leading to some extent within certain questions and may have influenced the responses given. Any benefits or undesired symptoms reported by participants may be attributable to other factors. Despite these limitations we believe this study presents a unique patient insight into the experiences of receiving healing therapy and the possible impacts on both the patient and their symptoms.

## Additional files

Additional file 1:
**The Adapted Individual Interview Schedule Version 2.**
Additional file 2:
**The Analysis Process.**
Additional file 3:
**Appendix B (online only).**

## Abbreviations

UK: United Kingdom
NHS: National Health Service
IBS: Irritable bowel syndrome
IBD: Inflammatory bowel disease
CD: Crohn’s disease
UC: Ulcerative colitis
