# Erratum to: Experiences of healing therapy in patients with irritable bowel syndrome and inflammatory bowel disease

**DOI:** 10.1186/s12906-015-0798-x

**Published:** 2015-09-15

**Authors:** Andrew Soundy, Rhonda T. Lee, Tom Kingstone, Sukhdev Singh, Pankaj R. Shah, Sandy Edwards, Lesley Roberts

**Affiliations:** School of Sport, Exercise and Rehabilitation Sciences, University of Birmingham, Birmingham, B15 2TT UK; Integrated Medicine Department, Freshwinds Charity, Prospect Hall, 12 College Walk, Selly Oak, Birmingham, B29 6LE UK; Department of Gastroenterology, Consultant in Gastroenterology, Good Hope Hospital, Sutton Coldfield, UK; Primary Care Clinical Sciences, School of Health and Population Sciences, University of Birmingham, Edgbaston, Birmingham, B15 2TT UK; Healer Member of The Healing Trust, Northampton, UK

Unfortunately, the original version of this article [1] contained an error. Sandy Edwards was not listed among the authors. The correct version can be found below.

Soundy, A.^1^, Lee, R T.^2^, Kingstone, T.^2^, Singh, S.^3^, Shah, P R.^2^, Edwards, S^5^., Roberts, L.^4^

^1^School of Sport, Exercise and Rehabilitation Sciences, University of Birmingham, Birmingham B15 2TT, UK

^2^Integrated Medicine Department, Freshwinds Charity, Prospect Hall, 12 College Walk, Selly Oak, Birmingham B29 6LE, UK

^3^Department of Gastroenterology, Consultant in Gastroenterology, Good Hope Hospital, Sutton Coldfield, UK

^4^Primary Care Clinical Sciences, School of Health and Population Sciences, University of Birmingham, Edgbaston, Birmingham B15 2TT, UK

^5^Healer Member of The Healing Trust, UK
