# Supplementary material for: Experiences of healing therapy in patients with irritable bowel syndrome and inflammatory bowel disease
Source: BMC Complement Altern Med. 2015 Apr 3;15:106. doi: 10.1186/s12906-015-0611-x (PMC4391663; doi:10.1186/s12906-015-0611-x)
Supplement: Additional file 1: — The Adapted Individual Interview Schedule Version 2. [file 12906_2015_611_MOESM1_ESM.docx]

Supplementary file A: The Adapted Individual Interview Schedule Version 2

Bio-psychosocial Impact of Illness

Could you describe how the symptoms began?

After the symptoms began could you tell me how your illness progressed and changed?

What aspects of your life are most affected by your symptoms? Prompt: Family Life, Social Life and Work Life

What symptom has affected your life the most?

What is the most important thing for you to change? Prompt: Symptom to change, impact on life.

Are there factors in your life that you associate with your symptoms?

Is there a time or place where the symptoms are worse?

Does your disease influence you personally?

If so, how?

*Prompts*

Does it impact on how you see yourself?

Do you find you have to make adaptations in your life to enable you to cope with your illness? Prompt if not raised: Diet, Exercise, Relaxation, Therapy

What approaches have you taken on to combat your illness?

Prompts: Diet, exercise, nutrition, alternative therapy

Preparing for experiencing and reflecting on healing

Did you understand what was meant by healing when you were invited on the trial?

What you were hoping from the therapy?

Did it achieve this?

How have you found the healing therapy?

Has it helped your symptoms? Which symptoms has it helped most?

What are the best and worst aspects of this therapy?

Do you think this therapy has influenced other aspects of your life? If yes in what way?

Has your view of healing changed since you began the trial?

Is the relationship or connection you have with the therapist important?

Can you describe what happens during a session?

Can you describe the experience and your associated feelings? Prompt: Anxious / relaxed

Could you describe any physical sensations you experience or perceive?

Do you feel physically that your symptoms have changed **during** the session?

Have you felt differently in other ways **during** the session? Prompt: Anxious / relaxed

Do you feel you had the right number of sessions? Does healing affect you differently in different sessions? – Can you explain this?

Can you identify difference changes across the six healing sessions?

Bio-psychosocial impact and outcomes of healing

Is there any influence of healing in your life after the healing sessions?

In what way has healing benefited you? – Prompt – symptoms, other aspects of life

Did you experience any negative effects of healing? - Prompt – symptoms worsening, other physical symtoms (e.g. headache), other negative impacts e.g. on relationships etc.

Would you consider more healing sessions? If you would why?
