# Supplementary material for: Experiences of healing therapy in patients with irritable bowel syndrome and inflammatory bowel disease
Source: BMC Complement Altern Med. 2015 Apr 3;15:106. doi: 10.1186/s12906-015-0611-x (PMC4391663; doi:10.1186/s12906-015-0611-x)
Supplement: Additional file 2: — The Analysis Process. [file 12906_2015_611_MOESM2_ESM.docx]

Supplementary File B: The Analysis Process

Techniques used:

1. Commenting on interviews. The primary author commented on interviews as did key members of the team. Comments took the form illustrated below. This illustrated target comparison very early on of concepts of interest.

AS: ….what is the value of healing if you had to describe it to someone else, what would you say to them?

PF2: i think the positive, what would you call it? I think just having a positive outlook and a calm, a very calm demeanour, very relaxing approach, umm, the full contact, you know, that wont suit everyone because it is very personal, but she also made it personal to me, I mean she cradled my head at one point and that felt really nurturing and she [therapist] said, well that’s not, not necessarily what we are taught as a standard, but felt for your you, that is something i thought you would benefit from, appreciated and i did and that is probably why some of the sessions were quite emotional really. And even a touching of hand, umm, she sort of place her hand to different parts you know, shoulders hands and i found that really reassuring and really calming.

1. Idea webbing used by Dr Soundy, below is a small example of an idea web very early on in the process. This was used to help develop analytical codes and take the analysis beyond a descriptive approach.

1. Finally the fragmenting process took place over several revisions of the analysis. This processes involved several authors critiquing the names of the codes, the structure and the content of interviews. The thematic development is placed in date order and only reflects the patients understanding of their condition and symptoms to illustrate changes made and processes involved.

10/05/2011

| **Theme** | **Sub-theme Break down** | **Code** | | **Participant Data** |
| --- | --- | --- | --- | --- |
| Understanding the disease | Control | Ruling their life | “because it was totally, umm, consuming me and ruling what i did” (PF4) | |
|  | Affects activities | In the house | “it causes issues in the house because obviously we have one toilet” (PF4) | |
|  |  | Social activities | “Whereas I have gone a bit within myself, locked myself away, a little bit, not wanting to socialise and stuff” (PF4) | |
|  | Talking about it | Avoid talking about it | “i hide a lot, people say are you ok PF4 and i say im fine, but i am really not” (PF4) | |
|  |  | Treat it as a joke | “I have sort of changed it into a bit of a joke” (PF4) | |
|  | Physical | Appearance changes | “I would look like i am 6 months pregnant” (PF5)  “you haven’t got the energy, you feel like shutting yourself away because you’re getting bigger” (PF6) | |
|  | Psychological | Emotions | “it does affect you psychologically” (PF5) | |

26/05/2011

Table 1 Considering patients understanding of illness and healing

| **Perception of Healing** | No idea | Don’t know what it is | “it wasn’t something i had or would have considered before” (PM1)  "I didnt really know what to expect before" (PF4) |
| --- | --- | --- | --- |
|  |  | Don’t know what to expect from it | “I didn’t know what to expect” (PF1)  “I didn’t know what to expect out of it” (PF1) |
|  | Mixed understanding | Stereotypes | “all I know about it was that it was a wishy washy ancient art...the closes reference i have to it...[is] these kind of healing shops” (PM1) |
|  | Recommended | Doctor told me | “”he said there was something in it” (PM1) |
|  | Just a more positive mind | Something you can do yourself | “”my whole idea of this thing is that actually you are healing yourself, a positive state of mind” (PM1) |
|  | Healer says | Something else | “healer is actually doing it physically, changing something” (PM1) |
| **Influence of illness on life domains** | **Stress** | Pressured environments | "stress related around college work and exams at school" (PF1)  “When I am upset or stressed my stomach has always been affected” (PF4) |
|  |  | Lifestyle | "my accelerated lifestyle has made a big impact on my heath" (PF2) "I was member of a cabin crew...irregular sleeping eating patterns, unique stressful environment" (PF2) |
|  |  | Worry | "The thought of going back to work frightened me, because i am unreliable, not because i don't care, because every day is different" (PF4) |
|  | **Diet** | Change of diet | "I had prepared a lamb casserole....I wasn't intending to eat [but i did]" (PF2)  “the whole diet thing is very complex” (PM1)  "I do need nutrition based advice to completely eliminate really anything that might aggravate" (PF2) |
|  | **Psychological make up** | Thinking and analysing too much | "I'm a thinker, analyser, and i have had quite as stressful life, been through quite a lot, so that, doesn't help my problems" (PF4)  "I just tie myself in knots, it just aggravates the situation" (PF4) |
|  | **Control** | **Ruling their life** | “because it was totally, umm, consuming me and ruling what i did” (PF4)  “used to self medicate for a long time” (PM1) |
|  | **Affects activities** | **In the house** | “it causes issues in the house because obviously we have one toilet” (PF4) |
|  | **Talking about it** | **Avoid talking about it** | “i hide a lot, people say are you ok PF4 and i say im fine, but i am really not” (PF4) |
|  |  | **Treat it as a joke** | “I have sort of changed it into a bit of a joke” (PF4) |
|  | **Physical** | **Appearance changes & social withdrawal** | “I would look like i am 6 months pregnant...it does affect you psychologically” (PF5)  “Whereas I have gone a bit within myself, locked myself away, a little bit, not wanting to socialise and stuff” (PF4)  “you haven’t got the energy, you feel like shutting yourself away because you’re getting bigger” (PF6) |
|  | **Self Help** | **Searching** | "you are willing to try anything" (PF4)  “I am going down many many paths to do this” (PM1) |
|  |  | **Relaxing through exercise** | "swimming helps me...I come out feeling very relaxed and actually sleep very well" (PF2)  Once i had a go in the hydrotherapy building, i had a go in the pool...that wa quite good" (PM2) |

22/11/2012

Table 1 Bio-psychosocial impact of illness on patient

|  | **Attributed causes of illness** | Pressured environments | "stress related around college work and exams at school" (PF1 - IBS)  “When I am upset or stressed my stomach has always been affected” (PF4) |
| --- | --- | --- | --- |
|  |  | Job | "my accelerated lifestyle has made a big impact on my heath" (PF2 - IBS) "I was member of a cabin crew...irregular sleeping eating patterns, unique stressful environment" (PF2 - IBS) |
|  | **Psychological impact** | Thinking and analysing too much | "I'm a thinker, analyser, and i have had quite as stressful life, been through quite a lot, so that, doesn't help my problems" (PF4 - UC)  "I just tie myself in knots, it just aggravates the situation" (PF4 - UC) |
|  |  | Worry | "The thought of going back to work frightened me, because i am unreliable, not because i don't care, because every day is different" (PF4 - UC) |
|  |  | Controlling/ruling their life | “because it was totally, umm, consuming me and ruling what i did” (PF4 - UC) |
|  |  | Appearance changes & social withdrawal | “I would look like i am 6 months pregnant...it does affect you psychologically” (PF5 - IBS)  “AS: does it affect your confidence? PF5-IBS: oh, it blows it to bits...you feel like shutting yourself away because you are getting bigger and bigger.”  “Whereas I have gone a bit within myself, locked myself away, a little bit, not wanting to socialise and stuff” (PF4 - UC)  “you haven’t got the energy, you feel like shutting yourself away because you’re getting bigger” (PF6 - IBS) |
|  |  | Impact on life | “it causes issues in the house because obviously we have one toilet” (PF4 - UC)  “it has messed up my life last year” (PM3 - UC) |
|  | **Social impact** | Avoid talking about it | “i hide a lot, people say are you ok PF4 and i say im fine, but i am really not” (PF4 - UC) |
|  |  | Treat it as a joke | “I have sort of changed it into a bit of a joke” (PF4 - UC) |
|  |  | Relaxing through exercise | "swimming helps me...I come out feeling very relaxed and actually sleep very well" (PF2 - IBS)  Once i had a go in the hydrotherapy building, i had a go in the pool...that was quite good" (PM2 - UC) |
|  | **Physical impact** |  |  |
|  |  | Change of diet | “the whole diet thing is very complex” (PM1 - IBS)  "I do need nutrition based advice to completely eliminate really anything that might aggravate" (PF2 - IBS) |
|  |  | Experiencing pain | "I had prepared a lamb casserole....I wasn't intending to eat [but i did]" (PF2 - IBS) |
|  |  | Sickness | “I was being sick more or less after each meal” (PF6 - IBS) |

23/01/2012

Table 1 Bio-psychosocial Impact of Illness on the Patient

|  | **Attributed causes of illness** | Pressured environments | "stress related around college work and exams at school" (PF1 - IBS)  “When I am upset or stressed my stomach has always been affected” (PF4) |
| --- | --- | --- | --- |
|  |  | Job | "my accelerated lifestyle has made a big impact on my heath" (PF2 - IBS) "I was member of a cabin crew...irregular sleeping eating patterns, unique stressful environment" (PF2 - IBS) |
|  | **Psychological impact** | Thinking and analysing too much | "I'm a thinker, analyser, and i have had quite as stressful life, been through quite a lot, so that, doesn't help my problems" (PF4 - UC)  "I just tie myself in knots, it just aggravates the situation" (PF4 - UC) |
|  |  | Worry | "The thought of going back to work frightened me, because i am unreliable, not because i don't care, because every day is different" (PF4 - UC)  “I am a born worrier anyway, it’s very difficult to change your mindset” (PF8 – IBS)  “I am upset and worried” (PF8 – IBS) |
|  |  | Controlling/ruling their life | “Because it was totally, umm, consuming me and ruling what I did” (PF4 - UC)  “What I am having difficulty with, because there doesn’t seem to be a pattern” (PF8 – IBS) |
|  |  | Appearance changes & social withdrawal | “I would look like i am 6 months pregnant...it does affect you psychologically” (PF5 - IBS)  “AS: does it affect your confidence? PF5-IBS: oh, it blows it to bits...you feel like shutting yourself away because you are getting bigger and bigger.”  “Whereas I have gone a bit within myself, locked myself away, a little bit, not wanting to socialise and stuff” (PF4 - UC)  “you haven’t got the energy, you feel like shutting yourself away because you’re getting bigger” (PF6 - IBS) |
|  |  | Impact on life | “it causes issues in the house because obviously we have one toilet” (PF4 - UC)  “it has messed up my life last year” (PM3 - UC)  “How [does] it can control me? I wouldn't go out, I would just stop in me four walls” (PF7 – UC) |
|  | **Social impact** | Avoid talking about it | “i hide a lot, people say are you ok PF4 and i say im fine, but i am really not” (PF4 - UC) |
|  |  | Treat it as a joke | “I have sort of changed it into a bit of a joke” (PF4 - UC) |
|  |  | Relaxing through exercise | "swimming helps me...I come out feeling very relaxed and actually sleep very well" (PF2 - IBS)  Once i had a go in the hydrotherapy building, i had a go in the pool...that was quite good" (PM2 - UC) |
|  | **Physical** |  |  |
|  |  | Change of diet | “the whole diet thing is very complex” (PM1 - IBS)  "I do need nutrition based advice to completely eliminate really anything that might aggravate" (PF2 - IBS) |
|  |  | Experiencing pain | "I had prepared a lamb casserole....I wasn't intending to eat [but i did]" (PF2 - IBS) |
|  |  | Sickness | “I was being sick more or less after each meal” (PF6 - IBS) |

31/01/2012

Table 1 Bio-psychosocial Impact of Illness on the Patient

|  | **Attributed causes of illness** | Pressured environments | "stress related around college work and exams at school" (PF1 - IBS)  “When I am upset or stressed my stomach has always been affected” (PF4 - UC)  “I get apprehensive...especially if I go somewhere new, or somewhere like an interview” (PF7 – UC)  “when you get stressed and uptight and that does affect it” (PF10-IBS) |
| --- | --- | --- | --- |
|  |  | Job | "my accelerated lifestyle has made a big impact on my heath" (PF2 - IBS) "I was member of a cabin crew...irregular sleeping eating patterns, unique stressful environment" (PF2 - IBS) |
|  | **Psychological impact** | Thinking and analysing too much | "I'm a thinker, analyser, and i have had quite as stressful life, been through quite a lot, so that, doesn't help my problems" (PF4 - UC)  "I just tie myself in knots, it just aggravates the situation" (PF4 - UC) |
|  |  | Drained/Tired | “its kind of like took its affect on me mentally and i feel drained, emotionally exhausted, and...I hear people try and tell me their problems and I think oh shut up” (PF11-UC)  “just wanted to go to bed for a couple of days” (PF8-IBS)  “I used to be feeling down all the time...I know you cant get rid of that” (PF12 – IBS). |
|  |  | Worry / anxiety | "The thought of going back to work frightened me, because i am unreliable, not because i don't care, because every day is different" (PF4 - UC)  “I am a born worrier anyway, it’s very difficult to change your mindset” (PF8 – IBS)  “I am upset and worried” (PF8 – IBS)  “I tend to worry a lot...I don’t find it easy to relax” (PF10-IBS)  “panic attacks and anxiety” (PF11-UC)  “I am a constant worrier, and if i haven’t got something to worry about I will generate something to worry about” (PM4 – UC) |
|  |  |  | “Because it was totally, umm, consuming me and ruling what I did” (PF4 - UC)  “The pain was pretty bad but I could cope with that...I would pass wind...and some would actually come out” (PF9 – UC) |
|  |  | Uncertainty of when it will strike | “What I am having difficulty with, because there doesn’t seem to be a pattern” (PF8 – IBS)  “I do find myself in a situation where I can be happy and relaxed...and all of a sudden, i got terrible pain and an onset of diarrhoea” (PF8 – IBS)  “i am not sure whether it kicks in when i am in a stressful situation...if my UC started up then I worry about it” (PM4 – UC) “I am not sure about...why it kicks in?” (PM4 – UC)  “it’s just unpredictable. It’s not predictable tomorrow or next week” (PF13 – UC) |
|  |  | Social perception | “I would look like i am 6 months pregnant...it does affect you psychologically” (PF5 - IBS)  “AS: does it affect your confidence? PF5-IBS: oh, it blows it to bits...you feel like shutting yourself away because you are getting bigger and bigger.”  “Whereas I have gone a bit within myself, locked myself away, a little bit, not wanting to socialise and stuff” (PF4 - UC)  “you haven’t got the energy, you feel like shutting yourself away because you’re getting bigger” (PF6 - IBS)  “I worked in a small office...if i had a funny expression on my face they would know” (PF9 – UC) |
|  | **Social impact** | Impact on life | “it causes issues in the house because obviously we have one toilet” (PF4 - UC)  “it has messed up my life last year..when it is bad i am on the toilet 23 hours of the day” (PM3 - UC)  “How [does] it can control me? I wouldn't go out, I would just stop in me four walls” (PF7 – UC)  “I had an English exam...I ended up going to the toilets a couple of times [before]” (PF7 – UC)  “I did get stomach cramps and it did get to the point where I couldn’t even go shopping...I was going to the toilet so much” (PF9 – UC)  “going out for a meal you feel is waste of time..at home I can eat what I know” (PF10-IBS)  “I first started getting symptoms of anxiety when I was 16...I would just get a feeling that everybody on the train knew...sometimes I couldn’t make the train journey” (PF11 – UC)  “the only thing I managed to do is my allotment” (PF11 UC)  “affected my work as well, I mean i sit on a till...but it really affected me breathing...I just got so fed up I just went back to GP” (PF12 – IBS)  “my employers don’t [take it into consideration]...they still don’t really understand, they want you in the hours to do the work” (P13 – UC)  “I hide a lot, people say are you ok PF4 and i say im fine, but i am really not” (PF4 - UC)  “I have sort of changed it into a bit of a joke” (PF4 - UC) |
|  |  |  |  |
|  |  |  |  |
|  |  | Relaxing through exercise | "swimming helps me...I come out feeling very relaxed and actually sleep very well" (PF2 - IBS)  “Once I had a go in the hydrotherapy building, i had a go in the pool...that was quite good" (PM2 - UC)  “I do yoga as well, it has helped me big time” (PF7 – UC) |
|  | **Physical** |  |  |
|  |  | Trying to relax more | “trying to relax more” (PF10-IBS) |
|  |  | Change of diet | “the whole diet thing is very complex” (PM1 - IBS)  "I do need nutrition based advice to completely eliminate really anything that might aggravate" (PF2 - IBS)  “[consultant] advised me to try exclusion of certain things [dietary]” (PF8 – IBS)  “I had to monitor my diet” (PF9 – UC)  “watching my diet” (PF10-IBS)  “I don’t have full fat milk...has helped me” (PF12 – IBS)  “I have found not eating white bread, doesn’t trigger it so much, it is dairy product, you know and things like that” (PF11 – IBS)  “know from very early days what sort of food to avoid” (PF13-UC)  “there is not a lot more I could do with my diet” (PM5 – IBS) |
|  |  | Avoiding food types: Experiencing pain | "I had prepared a lamb casserole....I wasn't intending to eat [but I did]" (PF2 - IBS)  “I have always felt stress straight in my stomach” (PF11- UC) |
|  |  | Historical symptoms: Physical appearance | “You could actually see my stomach coming out as if I was 6 months pregnant” (PF10-IBS)  “My stomach started blowing up and it was getting me all breathless and everything” (PF12 – IBS)  “I was starting to lose weight...I was admitted into hospital” |
|  |  | Historical Symptoms: fatigue | “If there was anything I could change it would be the fatigue” (PF13 – UC). |
|  |  | Historical symptoms: discharge | “I was being sick more or less after each meal” (PF6 - IBS)  “There was lots of blood and there was a kind of mucus there” (PF9 – UC)  “I have had things coming out of places they shouldn’t...I have had faeces coming out of my vagina then period out of my backside...affects everything, your femininity, your sexuality, how you feel on a day to day basis” (PF11-UC) |
|  |  | Medical Interventions | “I have got a stoma now so I haven’t got to rush to the toilet” (PF11-UC) |
|  |  | No symptoms | “When I started I was feeling great the best I have ever felt since starting the trial” (PF9 – UC) |

06/03/2012

Table 4 Bio-psychosocial Impact of Healing

| Theme | Sub-theme | Code | Example |
| --- | --- | --- | --- |
| Psychosocial Impact | Psychological traits | Worry anxiety | The thought of going back to work frightened me, because I am unreliable, not because I don't care, because every day is different" (PF4 - UC) “I am a born worrier anyway, it’s very difficult to change your mindset” (PF8 – IBS) “I am upset and worried” (PF8 – IBS) “I tend to worry a lot...I don’t find it easy to relax” (PF10-IBS) “panic attacks and anxiety” (PF11-UC) “I have always felt stress straight in my stomach” (PF11- UC) “I am a constant worrier, and if I haven’t got something to worry about I will generate something to worry about” (PM4 – UC) |
|  |  | Thinking | I'm a thinker, analyser, and I have had quite as stressful life, been through quite a lot, so that, doesn't help my problems" (PF4 - UC) "I just tie myself in knots, it just aggravates the situation" (PF4 - UC) |
|  | Sources of pressure | Work | "my accelerated lifestyle has made a big impact on my heath" (PF2 - IBS) "I was member of a cabin crew...irregular sleeping eating patterns, unique stressful environment" (PF2 - IBS) |
|  |  | Pressured environments | "stress related around college work and exams at school" (PF1 - IBS)  “When I am upset or stressed my stomach has always been affected” (PF4 - UC) “I get apprehensive...especially if I go somewhere new, or somewhere like an interview” (PF7 – UC) “when you get stressed and uptight and that does affect it” (PF10-IBS) |
|  | Illness uncertainty | Uncertainty of when it will strike | “What I am having difficulty with, because there doesn’t seem to be a pattern” (PF8 – IBS)  “I do find myself in a situation where I can be happy and relaxed...and all of a sudden, I got terrible pain and an onset of diarrhoea” (PF8 – IBS) “I am not sure about...why it kicks in?” (PM4 – UC)  “it’s just unpredictable. It’s not predictable tomorrow or next week” (PF13 – UC) “it often has a mind of its own” (F14-CD). |
|  | Outcome | Lifestyle restriction | “Because it was totally, umm, consuming me and ruling what I did” (PF4 - UC) “it causes issues in the house because obviously we have one toilet” (PF4 - UC) “it has messed up my life last year...when it is bad I am on the toilet 23 hours of the day” (PM3 - UC) “How [does] it can control me? I wouldn't go out, I would just stop in me four walls” (PF7 – UC) “I had an English exam...I ended up going to the toilets a couple of times [before]” (PF7 – UC) “I did get stomach cramps and it did get to the point where I couldn’t even go shopping...I was going to the toilet so much” (PF9 – UC) “going out for a meal you feel is waste of time..at home I can eat what I know” (PF10-IBS) “the only thing I managed to do is my allotment” (PF11 UC) “affected my work as well, I mean I sit on a till...but it really affected me breathing...I just got so fed up I just went back to GP” (PF12 – IBS) “my employers don’t [take it into consideration]...they still don’t really understand, they want you in the hours to do the work” (P13 – UC) “at the weekend I booked tickets for the theatre...I ended up sitting on my own [at the back near the exit for toilets]...it’s a battle all the time” (F15-IBS) “it’s always a bit of a management game every morning and I tend to stay around home most mornings. It is very rare that I would go out and not be close to home...it’s almost like having a stomach bug everyday” (F14-UC) |
|  |  | Withdrawal and embarrassment | “Whereas I have gone a bit within myself, locked myself away, a little bit, not wanting to socialise and stuff” (PF4 - UC) “The pain was pretty bad but I could cope with that...I would pass wind...and some would actually come out” (PF9 – UC) “you haven’t got the energy, you feel like shutting yourself away because you’re getting bigger” (PF6 - IBS) “I hide a lot, people say are you ok PF4 and I say I’m fine, but I am really not” (PF4 - UC). “the second time i came back [to a cinema seat], I thought well I can’t do this again, I will just have to leave” (F15-IBS) |
|  |  | Social confidence, perceptions and meta-perceptions | “I would look like I am 6 months pregnant...it does affect you psychologically” (PF5 - IBS) “I worked in a small office...if I had a funny expression on my face they would know” (PF9 – UC) “I first started getting symptoms of anxiety when I was 16...I would just get a feeling that everybody on the train knew...sometimes I couldn’t make the train journey” (PF11 – UC) “AS: does it affect your confidence? PF5-IBS: oh, it blows it to bits...you feel like shutting yourself away because you are getting bigger and bigger.” |
| Physical Impact | Historical Symptoms of Illness | Appearance | “You could actually see my stomach coming out as if I was 6 months pregnant” (PF10-IBS) “My stomach started blowing up and it was getting me all breathless and everything” (PF12 – IBS) “I was starting to lose weight...I was admitted into hospital” |
|  |  | Discharge | “I was being sick more or less after each meal” (PF6 - IBS)  “There was lots of blood and there was a kind of mucus there” (PF9 – UC) “I have had things coming out of places they shouldn’t...I have had faeces coming out of my vagina then period out of my backside...affects everything, your femininity, your sexuality, how you feel on a day to day basis” (PF11-UC) |
|  |  | Fatigue | “It’s kind of like took its affect on me mentally and I feel drained, emotionally exhausted, and...I hear people try and tell me their problems and I think oh shut up” (PF11-UC) “just wanted to go to bed for a couple of days” (PF8-IBS) “I used to be feeling down all the time...I know you can’t get rid of that” (PF12 – IBS).“If there was anything I could change it would be the fatigue” (PF13 – UC). |
|  |  | Pain | “I have always said pain is the most difficult one for me” (F14-CD) |
|  | Interventions | Exercise | "swimming helps me...I come out feeling very relaxed and actually sleep very well" (PF2 - IBS) “Once I had a go in the hydrotherapy building, I had a go in the pool...that was quite good" (PM2 - UC) “I do yoga as well, it has helped me big time” (PF7 – UC) |
|  |  | Diet | “the whole diet thing is very complex” (PM1 - IBS) "I do need nutrition based advice to completely eliminate really anything that might aggravate" (PF2 - IBS) “[consultant] advised me to try exclusion of certain things [dietary]” (PF8 – IBS) “I had to monitor my diet” (PF9 – UC) “watching my diet” (PF10-IBS) "I had prepared a lamb casserole....I wasn't intending to eat [but I did]" (PF2 - IBS)  “I don’t have full fat milk...has helped me” (PF12 – IBS) “I have found not eating white bread, doesn’t trigger it so much, it is dairy product, you know and things like that” (PF11 – IBS) “know from very early days what sort of food to avoid” (PF13-UC) “there is not a lot more I could do with my diet” (PM5 – IBS)  “I mainly stick to a low residue diet...I am extremely disciplined with what I eat” (F14-CD)  “I did say to [consultant] the list seems to be growing...so there is lots of things in foods [that cause symptoms problems” (F15-IBS) |
