# Supplementary material for: Experiences of healing therapy in patients with irritable bowel syndrome and inflammatory bowel disease
Source: BMC Complement Altern Med. 2015 Apr 3;15:106. doi: 10.1186/s12906-015-0611-x (PMC4391663; doi:10.1186/s12906-015-0611-x)
Supplement: Additional file 3: — Appendix B (online only). [file 12906_2015_611_MOESM3_ESM.docx]

**Appendix B (online only)**

**Key Features Identified Below**

- Patient was open to the experience and was expecting some positive response
- The patient was informed by therapists and understands that Reiki was or healing is associated with a detox and the undesired experiences are a natural product of the healing. This was a reason to attend following the first or subsequent undesired experiences.
- Burning pain around her head, feeling like her face was on fire within the session, pain occurred in most sessions, although stopped once therapist had reduced ‘the heat’.
- Following the session the patient identified a feeling like that of being poisoned with the effect of not going into work the following day. In addition the patient reported crying following session and feeling ill.
- The patient reported that the therapist themselves was very supportive and a good communicator who provided empathy and listened to the patient. This was a reason to attend following the first or subsequent undesired experiences.

**Key facts using verbatim quotes from the interview of P20 FIBS**

- *Beliefs about Healing Therapy*
  - Previous experience of Reiki^[[1]](#footnote-1)^ was that “I just cried the whole way through it, umm, and for no apparent reason” therapist explained to PF15 that “she just explained to me that, that was the Reiki working”
  - Open to the experience for instance PF15 stated “I don’t really understand the healing process I just kept this open mind” and “I wasn’t sure how it [healing therapy] would work for me now with the IBS...I went in with an open mind”.
  - From previous experience she stated that “I know that the channels of energy were meant to be cleared and things like that”
  - “I suppose healing in my head I thought would be a positive thing, I haven’t found that and I am not saying it’s wrong what has happened, because, I suppose its clearing out some of these blockages that are in my channels, I don’t know in some way or another. But it hasn’t been a pleasant experience.”
- *Feelings or Experiences of the Sessions*
  - First session: “felt so ill after the first session” “I was just cooking the dinner and I just felt very very strange and my [close relative] said P20F IBS, just go and sit down. I know why my head wasn’t working properly, it felt really woolly...the next say I still felt completely out of sorts” “felt hot” the next day
  - “I had this burning pain which I was saying to [therapist] about which was right in the back of my head, really really hot, it happened the first time she did it as well, very odd, really hot, umm, today I feel quite hot and the first time she did it I was really hot”
  - “I felt very strange with it, the whole thing”
  - “I didn’t expect to feel so bad” “I wasn’t expecting to feel so bad”
  - “I haven’t found it a pleasant experience at all”
  - “when I left the office the first time I went into the loo and I picked up a nice text from my daughter and I just burst into floods of tears, I hadn’t got emotional during the sessions, umm, just this heat, or essentially, she tailored it, a little bit better, not so intense, I haven’t felt like this, I feel [felt at the time] like oh my face is on fire at the moment.”
  - “I hadn’t felt anything really once she turned the heat down, it sounds a bit weird, but that’s how it felt to me.”
- *Interaction with the Therapist*
  - Context: valuing the therapist as an individual “[therapist name] has been very good”
  - Communication:
    - “After the first week I said to her, look I am here, but I don’t know if I am staying. Because I found I felt so ill after the first session...she [therapist name] did tell me that I should rest that evening. You know not try to do too much, drink, umm.”
    - “I explained it to the therapist in as much as I felt like kind of, it was like it was poison, I know that is a terrible work to use and it is to do with the detox she [therapist] explained to me afterwards, this is a sort of detox process, and I felt like that, that everything was all shifted but it wasn’t [a detox] it was all jumbled up somehow and in fact I had to change my day at work the next day because I still felt really hot as well. I think in hindsight I really did not drink enough, because I get dehydrated really easily”
    - “anyway the next week she said ok, we won’t be so intense this time, and she still did some of the Reiki with me, but I sat up and umm, she was talking to me and that is when she discovered things like, that I am concerned about”
    - “She, [the therapist] has been great; she has been very good in the sense in that she has, sort of, tried to explore the other things that are stressing me out. Umm, because I do think that, that is a big part of why I do get these stomach problems”
    - “[the therapist] did listen to that and she has tried to help me with some of that”

1. We recognise that Reiki is different from Healing therapy, however, for this patient the differences were not acknowledged. [↑](#footnote-ref-1)
